# Behavior of Silica Nanoparticles Synthesized from Rice Husk Ash by the Sol–Gel Method as a Photocatalytic and Antibacterial Agent

**DOI:** 10.3390/ma15228211

**Published:** 2022-11-18

**Authors:** A. Alhadhrami, Gehad G. Mohamed, Ahmed H. Sadek, Sameh H. Ismail, A. A. Ebnalwaled, Abdulraheem S. A. Almalki

**Affiliations:** 1 Department of Chemistry, College of Science, Taif University, P.O. Box 11099, Taif 21944, Saudi Arabia; 2 Chemistry Department, Faculty of Science, Cairo University, Giza 12613, Egypt; 3 Nanoscience Department, Basic and Applied Sciences Institute, Egypt-Japan University of Science and Technology, Alexandria 21934, Egypt; 4 Faculty of Nanotechnology for Postgraduate Studies, Cairo University, Sheikh Zayed Campus, Giza 12588, Egypt; 5 Zewail City of Science, Technology and Innovation, Giza 12578, Egypt; 6 Electronics & Nano Devices (END) Lab, Physics Department, Faculty of Science, South Valley University, Qena 83523, Egypt

**Keywords:** silica nanoparticles, rice husk ash, sol–gel method, photodegradation efficiency, antibacterial activity

## Abstract

Silica nanoparticles (SiO_2_ NPs) are one of the most well-studied inorganic nanoparticles for many applications. They offer the advantages of tunable size, biocompatibility, porous structure, and larger surface area. Thus, in this study, a high yield of SiO_2_ NPs was produced via the chemical treatment of rice husk ash by the sol–gel method. Characteristics of the prepared SiO_2_ NPs were validated using different characterization techniques. Accordingly, the phase, chemical composition, morphological, and spectroscopic properties of the prepared sample were studied. The average particle size of the SiO_2_ NPs was found to be approximately 60–80 nm and the surface area was 78.52 m²/g. The prepared SiO_2_ NPs were examined as photocatalysts for the degradation of methyl orange (MO) dye under UV irradiation. It was found that the intensity of the characteristic absorption band of MO decreased gradually with exposure time increasing, which means the successful photodegradation of MO by SiO_2_ NPs. Moreover, the antibacterial activity of obtained SiO_2_ NPs was investigated by counting the coliform bacteria in the surface water using the most probable number (MPN) index method. The results revealed that the MPN of coliform bacteria untreated and treated by SiO_2_ NPs was estimated to be 170 CFU/100 mL and 10 CFU/100 mL, respectively, resulting in bacterial growth inhibition of 94.12%.

## 1. Introduction

Nearly 50% of the dyes used in the textile industry are azo dyes (methyl orange (MO) as an example). The release of these compounds into the environment causes a series of issues [1]. The government’s rules require that hazardous or carcinogenic dye residues and their byproducts be removed from textile effluents [2]. Thus, numerous physical, chemical, and biological procedures, including chemical precipitation, filtration, adsorption, coagulation, and electrocoagulation, as well as biological degradation (biodegradation) and ozonation, are used to remove organic molecules from water [3,4]. Heterogeneous photocatalysis is an effective advanced oxidation process and offers a considerable advantage over other advanced oxidation processes, since it enables the transformation, minimization, and deactivation of persistent chemicals in the water, as well as mineralizing all pollutants (bacteria, viruses, dyes, organic soils, air pollution, etc.) [5,6]. This method involves producing a hole/electron pair in the presence of a photocatalyst by excitably exposing the organically contaminated water to artificial ultraviolet radiation (UV lamp) or natural (solar) [7].

Moreover, the photocatalysis process does not require the consumption or addition of chemicals except for some applications that include other oxidants such as hydrogen peroxide (H_2_O_2_) to enhance the efficiency of the photocatalytic process. Thus, photocatalysis has shown promising properties as one of the emerging technologies in the field of environmental applications such as water treatment and air purification [8].

In recent decades, nanomaterials have proven their potential to find sustainable and effective solutions to global issues [9]. These troubles include increased energy demand, resource depletion, and health issues [10,11]. Realistically, the effective ability of these particles to generate driving forces for physical and chemical processes is associated with the various local surroundings of atoms, which are uncovered on the solid surfaces as compared to those in the bulk [12,13]. Therefore, the specific surface area of nanomaterials increases due to the small particle sizes [14]. This means the nanomaterials are more effective and lead to improving activity in related applications [15,16]. Thus, the surface area of nanomaterials plays a major role in the activity of these materials [17,18].

Because of their superior thermal properties at high temperatures and pressures, strong biocompatibility, low toxicity, simple synthetic approach, and widespread synthetic availability, SiO_2_ nanoparticles are indeed one of the significant materials for biological and industrial applications. Additionally, SiO_2_ nanoparticles are widely employed in a variety of different industries, including those that utilize energy sources, sensors, and catalysis [19,20]. Moreover, silica nanoparticles are inert and have good chemical stability. They also exhibit a remarkable specific surface area, significant capacity for organic material adsorption, and high surface porosity [21,22,23].

Badr et al. (2008) reported that for many processes, silica is essentially inert; however, it has observable activity toward some catalysts. Pure silica has been demonstrated to inhibit photocatalytic processes such as the photoepoxidation of propene, photo-metathesis of propene, and photo-oxidation of CO when exposed to ultraviolet (UV) radiation. Silica-based photocatalysts such as silica–alumina, silica-supported zirconia, and silica–alumina–titania have been employed and show activity when exposed to UV light at room temperature. Sol–gel preparation of SiO_2_ resulted in the formation of surface photoactive sites, which were identified by vacuum-ultraviolet/ultraviolet spectroscopy (VUV-UV), Fourier transform infrared spectroscopy (FTIR), electron paramagnetic resonance (ESR), and photoluminescence spectroscopy. The Si-O^−^ nonbridging bond’s IR symmetric stretching vibration appeared at 950 cm^−1^. In addition, a transformation from SiO_2_ structural units (a three-dimensional network structure) to SiO_4_ (isolated tetrahedron) structural units occurred by the increase in peak intensity at 950 cm^−1^, which means an increase in the amount of nonbridging oxygen. Thus, four Gaussian peaks at roughly 2.5, 2.8, 3.0, and 3.2 eV were visible in the photoluminescence spectra of silica when the SiO_2_ NPs were photoexcited by UV light at wavelengths below ~390 nm, which corresponds to the charge transfer (258 nm wavelength) from the bonding orbital of Si-O to the *2p* nonbonding orbital of nonbridging oxygen.

The self-trapped exciton’s disintegration into a pair of holes localized on oxygen and electrons localized on nearby silicon in the silica matrix is what causes the luminescence band between 2.5 and 2.8 eV. As opposed to this, the bands at 3.0 and 3.2 eV are produced by electron–hole recombination at unidentified oxygen-deficient-associated defect sites [24]. Accordingly, these features have become attractive factors for the application of these SiO_2_ NPs as a photocatalyst in various applications such as pharmaceuticals, chemical sensors, and water treatment [25]. Particularly, photocatalytic nanoparticles have revolutionized conventional water treatment processes, as the oxidants produced by these photocatalysts are strong enough to degrade the organic dyes and inactivate pathogenic microorganisms in the water [21,26].

Rice husk (RH) is a major agricultural waste that can be used as fuel. Soil properties and agricultural variables are used to affect the chemical composition of RH, including lignin, hemicellulose, cellulose, and minerals [27,28]. Rice husk ash is a major resource that is free of cost and in nearly sustainable abundance, and thus many researchers have become interested in how this industrial waste can be used. Rice husk ash (RHA) usually contains more than 60% silica (SiO_2_), 10–40% carbon, and other minor mineral compositions [29]. Table 1 shows the chemical constituents of RHA as reported in much of the literature.

Currently, many recent technologies are used to convert agricultural waste into renewable energy. The conversion of biomass into energy products can be performed through two processes: (1) biochemical process, and (2) thermochemical process [30]. Therefore, the thermochemical process by which solid biomass is converted into a primarily liquid product called bio-oil is known as the rapid pyrolysis of biomass. Moreover, bio-oil production is accompanied by some production of valuable by-products such as noncondensable gases and solid biochar. Solid residues, gases, and bio-oil are often produced permanently, the proportion of which varies greatly depending on the process parameters. In the case of rice husk, the minerals represent approximately 20% of the initial biomass, especially silica [31]. Since RHA contains a high proportion of silica, it thus has the potential to be a commercially feasible raw material for the production of silicate and silica materials [32,33]. Accordingly, using the rice husk as a source of silica nanoparticles could be a more economically and environmentally friendly option.

**Table 1:** The chemical composition of RHA from the literature indicates that silica is the major constituent of rice husk ash.

Chemical Constituents	Percentage Composition (%)			
	Raheem et al. [34]	Jongpradist et al. [35]	Rambo et al. [36]	Rama Subbarao et al. [37]
SiO_2_	86.51	93.00	91.40	91.10
Al_2_O_3_	0.61	0.17	0.48	0.40
Fe_2_O_3_	0.60	0.35	0.02	0.40
CaO	0.71	0.91	0.50	0.40
MgO	1.53	0.42	0.35	0.50
SO_3_	0.02	0.11	---	0.10
Na_2_O	0.05	0.63	0.00	0.10
K_2_O	1.89	2.82	1.50	2.20
P_2_O_5_	4.20	---	0.18	---
TiO_2_	---	---	0.00	---
MnO	---	---	0.37	---
Loss of ignition (L.O.I)	3.88	4.70	5.63	4.80
SiO_2_ + Al_2_O_3_ + Fe_2_O_3_	87.72	93.50	91.90	91.90

Therefore, this study aimed to synthesize a silica nanoparticle (SiO_2_ NPs) from the rice husk ash by the sol–gel method. Then, the prepared SiO_2_ NPs were subjected to the appropriate characterization using X-ray diffraction (XRD), scanning electron microscopy (SEM), energy-dispersive X-ray spectroscopy (EDX), transmission electron microscopy (TEM), atomic force microscopy (AFM), BET surface area, and water-contact angle (CA) instruments. Afterward, the prepared SiO_2_ NPs underwent studying the optical properties. Furthermore, the photocatalytic performance of prepared SiO_2_ NPs towards the degradation of methyl orange (MO) dye in the colored water samples was investigated. Moreover, the antibacterial behavior of SiO_2_ NPs against coliform bacteria in the surface water samples was inspected.

## 2. Experimental Work

### 2.1. Materials

Rice husk was collected from agricultural fields in Giza, Egypt. Ammonia solution (NH_4_OH, AR, 18–20% purity, molecular weight of 35.05 g/mol) and hydrochloric acid (HCl, 35% purity, molecular weight of 36.46 g/mol) were obtained from Loba Chemie (Mumbai, India). Sodium hydroxide pellets (NaOH, ACS reagent, assay ≥ 97%, molecular weight of 39.99 g/mol) were obtained from Fisher Scientific (Waltham, MA, USA). Ethanol solution (C_2_H_5_OH, 96% purity, a molecular weight of 46.07 g/mol) and methyl orange powder (MO, C_14_H_14_N_3_NaO_3_S, ACS reagent, dye content of 85%, molecular weight of 327.33 g/mol) were obtained from the Merck group (Darmstadt, Germany). MacConkey Broth, PH EUR–USP medium for the detection of coliforms according to PH EUR (Broth Medium G–Harmonised) purchased from biolab for splendid isolation (Budapest, Hungary). All chemicals were used as received without purification. Double distilled water was used throughout all experiments.

### 2.2. Preparation of Silica Nanoparticles (SiO_2_ NPs)

Silica was produced from ash using the sol–gel method through simultaneous hydrolysis and condensation reaction. A sol of sodium silicate, silicon alkoxide, or halide gels was converted into a polymeric network of gel, where during silica synthesis by sol–gel process under certain conditions like the restriction of gel growth, silica gets precipitated. In such preparation, the steps involved are coagulation and precipitation from silica solution. Moreover, the synthesis process of silica gel by this method is known as xerogel. Firstly, the conversion of ash to silica gel involves the reaction of ash with caustic lye to produce sodium silicate. Then, is followed by the reaction of sodium silicate with hydrochloric acid to yield the silica [22,38,39,40]. Therefore, various methods are possible such as introducing an element from its alkoxide in an alcoholic solution or as a sol in an aqueous solution. For the same element, various alkoxides with varying-length organic chains (methoxide, ethoxide, propoxide, butoxide) can be utilized. For instance, in this case, the silicon methoxide hydrolyzes initially as a monomer, while ethoxide poly-condensates first, before completing the hydrolysis process [41,42].

**Figure 1:**
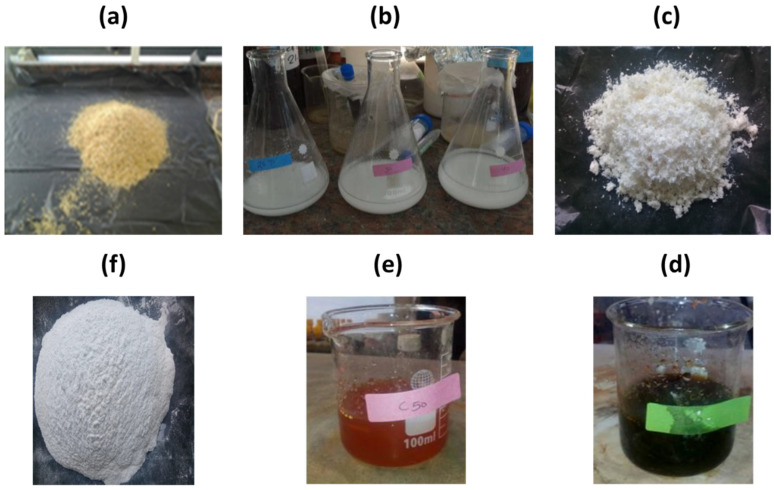
(**a**) Rice husk, (**b**) purification step, (**c**) purified RH, (**d**) RHA after the carbonization process, (**e**) formation of sol–gel, (**f**) final SiO_2_ NPs product.

The following steps were applied to obtain silica nanoparticles from rice husk ash: To remove the dust and other soluble organic and inorganic impurities, the rice husk was soaked and washed in distilled water. The washed rice husk was dried in an oven at 120 °C for 24 h. The dried rice husk was immersed in an acidic solution (HCl, 0.2 mol/L) for 24 h (Equation (1)) to dissolve the carbonate components, after which it was repeatedly rinsed with distilled water to remove the acid and then was air-dried for another 24 h. The purified rice husk was burned at 800 °C for 1 h in a muffle furnace. After that, 10 g of the burned rice husk ash was mixed with 2.5 M of NaOH. The mixture was then heated for 4 h before being filtered to obtain a colorless viscous solution. This solution was designated as Na_2_SiO_3_ stock solution (Equation (2)). Next, the Na_2_SiO_3_ solution was reacted with HCl to obtain the aqueous silica solution (Equation (3)). Subsequently, 1 g of the aqueous silica solution was mixed with 142.8 mL of ethanol, 20 mL of water, and 3.14 mL of ammonia solution, and stirred for 1 h at room temperature. The mixture was then mixed with a quaternary cationic ammonium surfactant followed by agitation for 4 h at room temperature. The mixture was left for 48 h to evaporate the solvent and form a gel. Finally, silica nanoparticles were produced by calcining the gel at 600 °C for 2 h (Equation (4)). Figure 1 displays photos of the production steps of SiO_2_ NPs from the rice husk.

**Figure 2:**
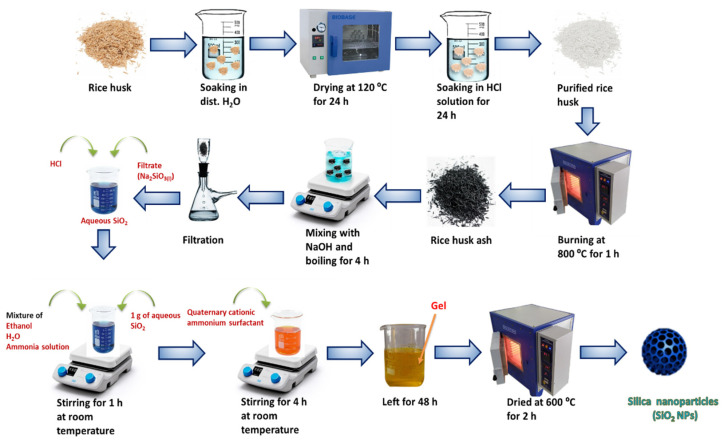
Schematic illustration for preparation of the silica nanoparticles from rice husk ash by sol–gel process.

The above steps could be represented by the following equations and the scheme shown in Figure 2.

(1) $${\mathrm{SiO}}_{2}{\left(\mathrm{RHA}\right)}_{\left(s\right)}+2{\mathrm{HCl}}_{\left(aq\right)}+H_{2}O_{\left(l\right)}\to H_{2}{\mathrm{SiO}}_{3_{\left(s\right)}}+2{\mathrm{Cl}}_{\left(aq\right)}^{-}+2H_{\left(aq\right)}^{+}$$

(2) $$H_{2}{\mathrm{SiO}}_{3}{}_{\left(s\right)}+2{\mathrm{NaOH}}_{\left(l\right)}\overset{\Delta }{\to }{\mathrm{Na}}_{2}{\mathrm{SiO}}_{3_{\left(l\right)}}+2H_{2}O_{\left(l\right)}$$

(3) $${\mathrm{Na}}_{2}{\mathrm{SiO}}_{3}{}_{\left(l\right)}+2{\mathrm{HCl}}_{\left(l\right)}\to {\mathrm{SiO}}_{2_{\left(aq\right)}}+2{\mathrm{NaCl}}_{\left(aq\right)}+H_{2}O_{\left(l\right)}$$

(4) $${\mathrm{SiO}}_{2_{\left(aq\right)}}\overset{C_{2}H_{5}\mathrm{OH},\;{\mathrm{NH}}_{4}\mathrm{OH}}{\underset{Quaternary\;ammonium\;cation}{⟶}}{\mathrm{SiO}}_{2}\mathrm{NPs}$$

The silica nanoparticle extraction yield (wt.%) was calculated using the following equation [43]:

Wt.% = (mass of silica nanoparticles/mass of used RHA) × 100(5)

Accordingly, the extracted yield of produced silica nanoparticles (wt.%) was calculated to be 29.1 wt.% SiO_2_.

### 2.3. Characterization Techniques

X-ray diffraction analyses were performed on the prepared SiO_2_ NPs using the Bruker D8 Discover instrument. The nanoparticles were scanned in a range of 2θ = 10–90° with a scan speed of 2°/min. The CuKα radiation source provided a wavelength of 0.154060 nm under an applied voltage of 40 kV and a current of 40 mA. The morphological structures and elemental composition of prepared nanoparticle surfaces were investigated at an acceleration voltage of 30 kV and various magnifications using a scanning electron microscopy (JEOL JXA-840A, Tokyo, Japan) analyzer equipped with an EDX analyzer. The morphology, shape, and size of the SiO_2_ NPs were examined using a transmission electron microscope (JEOL, TEM-2100, MA, USA) operating at a potential of 20 kV. A copper grid was sputtered by gold used to support the SiO_2_ NPs during the TEM investigations of the samples, where the SiO_2_ NP sample was sonicated in an ultrasonic cleaner (Elma, Singen, Germany) for 30 min after being diluted with distilled water. The coated copper grid was then covered with a few drops of the SiO_2_ NP sample, which was then allowed to dry at room temperature before TEM microscopy analysis was conducted. The SiO_2_ NPs were imaged in 2D topographic form using an atomic force microscope (AFM, 5600LS, Agilent, Santa Clara, CA, USA). The surface area of the produced SiO_2_ NPs was determined using Quantachrome’s NOVA touch LX2 model, NT2LX-2, USA. The SiO_2_ NP sample was degassed at 423 K for two hours before analysis. Using the N_2_ adsorption method at 77 K, the Brunauer–Emmett–Teller (BET), and Barrett–Joyner–Halenda (BJH) methods were used to assess the specific surface area and pore characteristics of SiO_2_ NPs, respectively. Finally, the wettability properties of synthesized SiO_2_ NPs were evaluated by an optical tensiometer, Theta Pulsating Drop contact angle analyzer manufactured by Biolin Scientific, USA. The optical properties of prepared SiO_2_ NPs and dye color removal were obtained using a computerized double-beam ultraviolet-visible spectrophotometer (SPECORD 200 PLUS, Analytik Jena, Jena, Germany) with 1 nm steps.

### 2.4. Optical Study of Synthesized SiO_2_ NPs

To examine the photocatalysis properties of the as-prepared SiO_2_ NPs, 50 mg of silica nanoparticles was dispersed in 150 mL of distilled water in a 200 mL beaker. To keep the suspension stable, it was gently stirred throughout the experiment and then exposed to the light of a UV lamp (mercury lamp) with a wavelength of 350 nm. Subsequently, 5 mL of the suspension was extracted at regular intervals to measure its optical properties.

### 2.5. Photocatalytic Study of Synthesized SiO_2_ NPs

To evaluate the photocatalytic activity of prepared SiO_2_ NPs under the effect of UV irradiation, methyl orange (MO) was used as a substrate. To monitor the photocatalytic degradation process, the optical absorption peak of MO at 465 nm was chosen. This characteristic absorption peak can be used to calculate the concentration of MO using the Lambert–Beer law. The experiment was carried out in the following manner: In a 200 mL beaker, 150 mL of 50 mg/L MO solution was prepared. To make a suspension, 50 mg of SiO_2_ NPs was dispersed in this solution. The catalyst concentration was kept constant for testing the dye concentrations vs. time. The suspension was magnetically stirred in the dark for 1 h to ensure that MO adsorption/desorption equilibrium was established on the surface of silica nanoparticles followed by irradiating the suspension with UV lamp light using a wavelength of 350 nm. To keep the suspension stable throughout the experiment, it was continuously and gently magnetically stirred. Then, 5 mL of the suspension was extracted at regular intervals times and centrifuged at 6000 rpm for 15 min to separate silica nanoparticles from the supernatant. A UV–Vis spectrophotometer was used to record time-dependent absorbance changes in the supernatant at wavelengths ranging from 190 to 1100 nm. The percentage of degradation was calculated using the following equation [44]:

(6) $$\mathrm{Degradation}\;\left(\%\right)=\frac{C_{0}-C_{t}}{C_{0}}\times 100$$

where C_0_ is the initial dye concentration and C_t_ is the dye concentration at a certain reaction time t (min). For comparison, a blank experiment was performed in the presence of the photocatalyst and the absence of UV light. Another experiment was carried out in the absence of a photocatalyst and the presence of UV light.

### 2.6. Microbial Study of Synthesized SiO_2_ NPs

The antibacterial activity of the obtained SiO_2_ NPs was investigated by counting the most probable number (MPN) index of coliform bacteria as colony-forming units (CFU) per 100 mL for canal water samples. To achieve this purpose, surface water samples were collected in sterile glass bottles and transported to the laboratory for further analysis according to the standard methods for the examination of water and wastewater, 23rd edition for 2017 [45]. To examine the antibacterial activity of SiO_2_ NPs against coliform bacteria using the most probable number, the following procedures were implemented according to the 4th edition incorporating the 1st addendum of the World Health Organization (WHO) guidelines for drinking water quality (GDWQ) [46].

The disinfection process was performed based on the direct contact between the contaminated water and the prepared SiO_2_ NPs, where the first sample was bacteriologically contaminated water without treatment by SiO_2_ NPs (control sample). Correspondingly, the second sample (150 mL) was mixed with 50 mg/L of SiO_2_ NPs under constant agitation (150 rpm) at room temperature for 90 min (SiO_2_ NP-treated sample). Subsequently, a filtration process was carried out on filter paper to separate the SiO_2_ NPs before the inoculation of samples. In the MPN method, 10 mL, 1 mL, and 0.1 mL of the samples were transferred to MacConkey fermentation tubes, where each water sample (without treatment and treated by SiO_2_ NPs) was typically collected in 5 double-strength tubes and 10 single-strength tubes. Five tubes containing 10 mL of double-strength MacConkey Broth media each received 10 mL of each water sample using a sterile pipette. Similarly, 1 mL of each water sample was inoculated into 5 tubes that had 10 mL of single-strength MacConkey Broth medium, and 0.1 mL of each water sample was added to the remaining 5 tubes that contained the same amount of single-strength MacConkey Broth medium. All tubes were incubated at 37 °C for 90 min. After incubation, each water sample was screened for the production of both acid and gas as an indication of a positive presumptive test. The acid production was detected by a color change and for the gas through the gas bubbles in the inverted Durham tube. Accordingly, the number of presumptive positive tubes was recorded. Then, the results of the presumptive test were compared with the MPN index standard chart, and the number of bacteria present in each tube was recorded to obtain the MPN/100 mL of total coliforms [47,48].

## 3. Results and Discussion

### 3.1. Characterization of Synthesized SiO_2_ NPs

**Figure 3:**
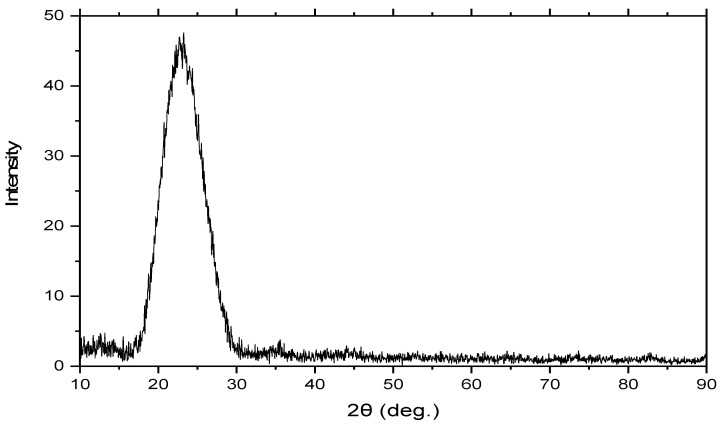
XRD diffractogram of synthesized SiO_2_ NPs from RHA.

The prepared SiO_2_ NPs were characterized using XRD, and the data obtained are represented in Figure 3. The XRD showed the absence of any other peaks related to impurities, and only the SiO_2_ phase was observed, which indicates that the majority of the prepared sample consists of SiO_2_. Furthermore, the silica nanoparticle diffractogram for the produced sample only shows the amorphous silica, which is distinguished by the presence of a single broad peak in the range of 2θ = 15–30°, that reaches its maximum intensity at 2θ = 22° (432), indicating a typical form for the amorphous nature of solids, therefore confirming the absence of any ordered crystalline structure. These observations agree with the results found in the literature [49,50,51]. Moreover, it was difficult to justify an accurate size of the SiO_2_ NPs because of their amorphous property. Thus, the Scherrer equation cannot be used to determine the crystallite size since the structure lacks any crystalline peaks.

**Figure 4:**
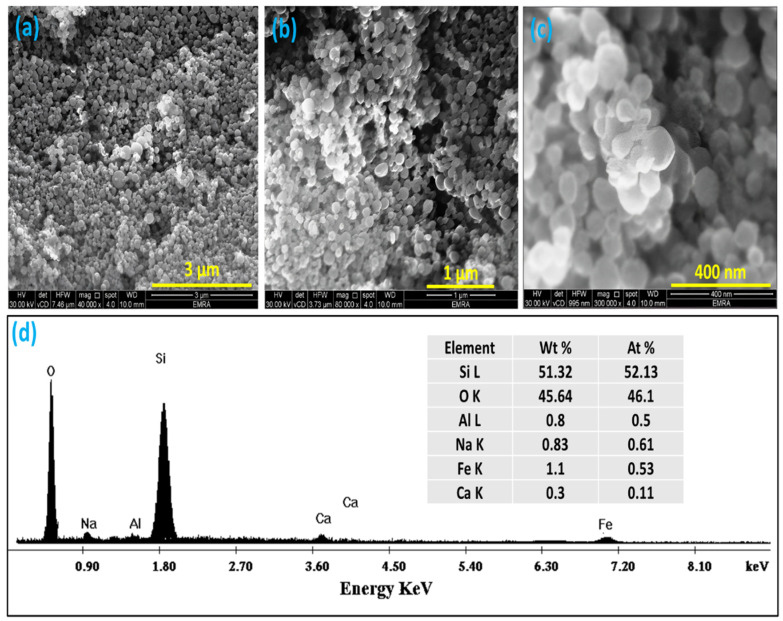
SEM images of the produced SiO_2_ NPs at different magnifications: (**a**) 40,000×; (**b**) 80,000×, and (**c**) 300,000×, respectively; (**d**) represent EDX spectrum and corresponding elemental table for the chemical composition of prepared SiO_2_ NP sample.

Figure 4a–c shows SEM images of the prepared SiO_2_ NPs with different magnifications. As shown in the images, it is easy to observe the spherical shape of prepared particles with sharp-edge structures. Silica nanoparticles exhibited excellent homogeneity in size and shape. In addition, the images showed that the prepared SiO_2_ NPs significantly formed in uniform nanoscale sizes. Moreover, the majority of as-obtained SiO_2_ NPs had average diameters of approximately 60–80 nm, which agrees with the data in previous studies [52,53].

The EDX analyzer was utilized to study the chemical composition of the prepared SiO_2_ NP sample. The obtained EDX spectrum and the corresponding table are presented in Figure 4d. The data illustrated in Figure 4d indicate that the existence of both Si and O elements in high concentrations reached 51.32 wt.% and 45.64 wt.% in the given sample, respectively, compared with the other elements such as Fe and Al, which exhibited a minor weight percent of about 1.1 wt.% and 0.8 wt.%, referring to the organic origin of the prepared SiO_2_ sample. These results confirm the phase purity of the prepared sample. Similar results were reported by Cendrowski et al. [54].

**Figure 5:**
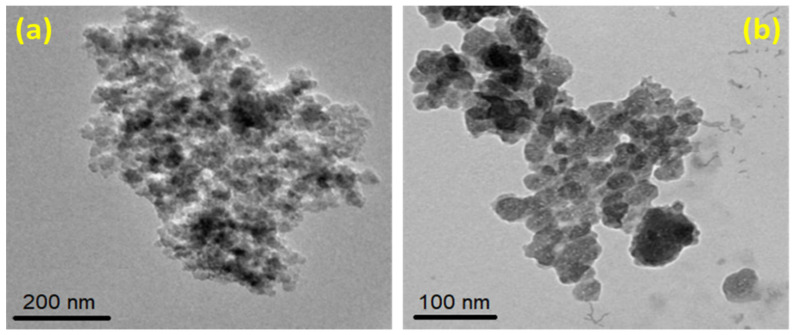
TEM images of SiO_2_ NPs depict the particle sizes at different scales: (**a**) 200 nm and (**b**) 100 nm, respectively.

Figure 5a,b show the different scale TEM images of produced SiO_2_ NPs. It could be observed that the SiO_2_ NPs are present in sizes of about 100 nm, with some aggregates. The high calcination temperature led to the desorption of the ammonium surfactant molecules that had adsorbed on the surface of SiO_2_ NPs during the preparation procedure, which resulted in a lower distribution of SiO_2_ NP states and aggregation between the particles via a Si-O-Si bridge. However, the use of a surfactant agent helped in improving the interfacial adhesion between the particles, causing decreases in the agglomeration and enhancing the particles’ dispersion. Le et al. reported a similar case [55].

**Figure 6:**
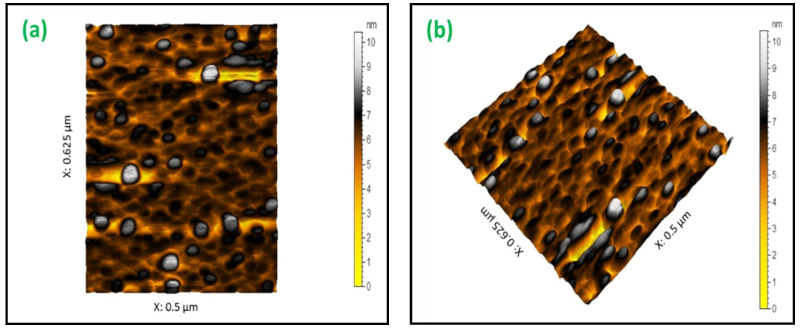
(**a**,**b**) AFM topographical images of the synthesized SiO_2_ NPs from RHA.

The produced SiO_2_ NPs particles’ surfaces were scanned by atomic force microscopy (AFM) imaging. The characteristic topography is provided in Figure 6a,b. The spherical shape and the homogeneous distribution of prepared SiO_2_ NPs could easily be observed. In addition, the particles’ surface appeared fairly roughened. The SiO_2_ NPs displayed a submicrometer-scale roughened surface, with a roughness value of 6.77 nm (root mean square, RMS) due to the agglomerated structures of individual nanoparticles. Generally, the results derived by AFM analysis well match those received from SEM and TEM analyses.

**Figure 7:**
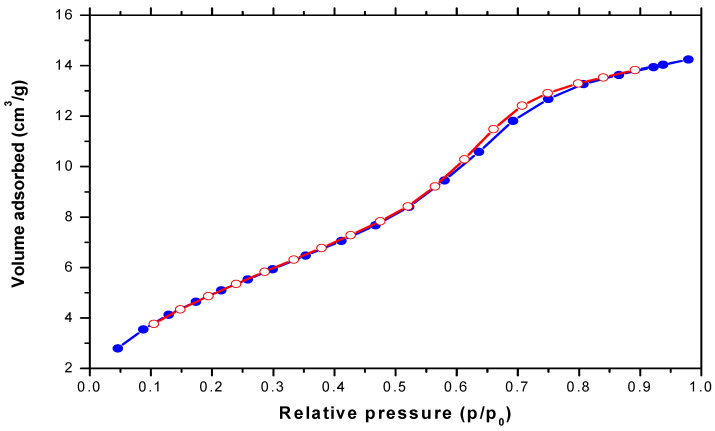
Nitrogen adsorption–desorption isotherm of the prepared SiO_2_ NPs, the blue line represents the adsorption branch, and the red line refers to the desorption branch of the isotherm.

The surface area, pore size, pore volume, pore structure, and porosity of produced SiO_2_ NPs were studied using the liquid nitrogen adsorption–desorption isotherm (Figure 7). According to the IUPAC nomenclature, the isotherm curve for the synthesized SiO_2_ NPs has a typical IV shape and an H1-type hysteresis loop, which is indicative of the material’s mesoporosity. The aforesaid results imply that the silica nanoparticles had a porous structure since the SiO_2_ NPs are proposed to include cylindrical pores based on their geometry. Generally, it is observed that the adsorbed gas volume tends to systematically and gradually increase as the condensation pressure increases, and conversely, the desorbed gas volume tends to systematically and gradually decrease as the gas pressure decreases, which provides strong evidence that adsorption–desorption branches of the isotherm are suitable for the PSD calculations. According to Table 2, the surface area of SiO_2_ NPs was found to be 78.52 m²/g, and the BJH cumulative surface area exhibited a good agreement between the adsorption and desorption branches values of the surface area of 40.63 m^2^/g and 38.48 m^2^/g, respectively, whereas the total pore volume was estimated as 0.062 cm^3^/g and the average pore diameter was about 3.158 nm. Previous studies mentioned similar results [56,57,58].

### 3.2. Wettability of Synthesized SiO_2_ NPs

**Figure 8:**
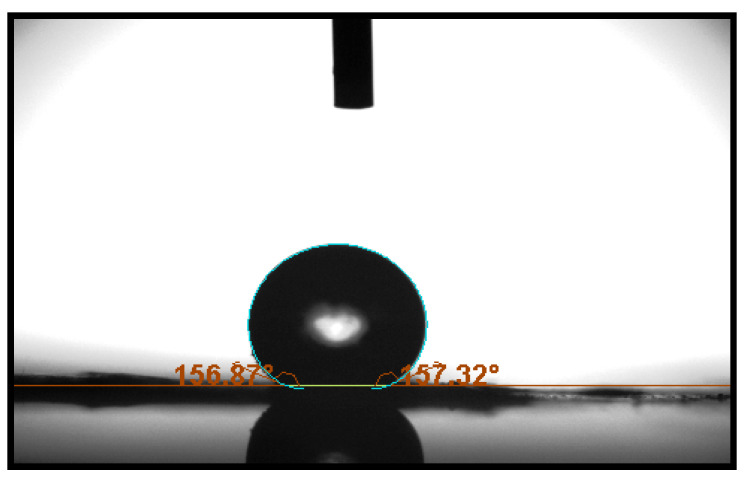
Water-contact image and numerical contact angle values of synthesized SiO_2_ NPs.

The water-contact angle of the prepared SiO_2_ NP sample was scrutinized in order to investigate the hydrophobicity of the SiO_2_ NPs. The produced sample was hydraulically pressed into a disc shape to observe the water-contact angle. As seen in Figure 8, SiO_2_ NPs showed significant large water-contact angles (mean), reaching 159.15° (left side) and 157.67° (right side) with a surface tension of about 76.6 [mN/m], demonstrating the superhydrophobic properties of the synthesized SiO_2_ NPs where the synthesized SiO_2_ NPs showed a water contact angle (CA) higher than 150° (150° < θ < 180°). It has been suggested that the long series (tail) of the silanol groups on the SiO_2_ NPs surface, which supply water-repelling properties to the surface of SiO_2_ NPs, are the main source of the hydrophobicity of SiO_2_ NPs. The water-wettability properties of the synthesized SiO_2_ NPs are summarized in Table 3.

### 3.3. Optical Properties

**Figure 9:**
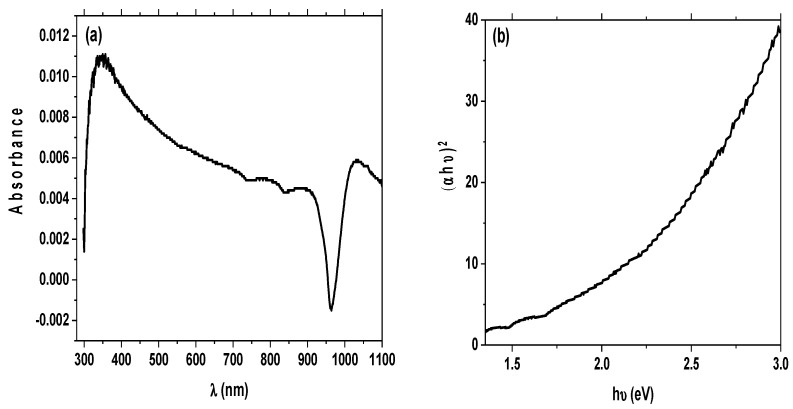
(**a**) Room-temperature UV–visible absorption spectrum of the prepared silica nanoparticles; (**b**) plot of (αhν)^2^ versus (hν) for the prepared synthesized SiO_2_ NPs nanoparticles.

Figure 9a shows the room-temperature UV–visible absorption spectrum of produced SiO_2_ NPs in the region of 300–1100 nm. As shown in the presented spectrum, there were no peaks observed for SiO_2_ NPs. As expected, since the sample primarily consists of SiO_2_, it is transparent to UV–Vis light in general. The very low absorbance values are justified by the possibility that contaminants, which are present in the sample in very low concentrations, are responsible for the weak absorption peak observed at about 320 nm. Consequently, the presence of some impurities such as Fe and Al oxides attached to the surface of the SiO_2_ NPs sample might contribute to the establishment of the absorption band. The SiO_2_ NPs were prepared from rice ash, which contained a tangible amount of these metal oxides. Thus, the presence of impurities can be considered when designating the band gap and photodegradation processes.

Since crystalline silica has a very large band gap (about 9 eV), it is transparent to UVB, UVA, and visible radiations. Accordingly, for enhancing both stability and delivery, it has been used to entrap dyes and drugs. Nevertheless, the existence of structural defects together with the decreased crystallinity can lower the band gap energy or increase the capacity of the material for UV photoactivation. Silica has a surface that is extremely reactive and capable of adsorbing both organic and inorganic molecules due to the presence of silanol groups. This property can be improved when silica is formed into colloidal particles. Thus, silica nanoparticles have detectable photocatalytic activity under UV illumination [59]. Moreover, amorphous silica nanoparticles have been examined and characterized for many defects, including nonbridging oxygen hole centers, neutral deficient oxygen centers, and impurities/defect states in silica or centers such as oxygen vacancies. However, the existence of structural defects makes silica photoactivable with UV radiation. These defects exhibited optical absorption, which covers the UVB and UVA ranges and improves the catalytic capabilities of SiO_2_ NPs under UV irradiation [60].

These structural defects can be divided into paramagnetic and nonparamagnetic defects such as E’ centers based on their ability to absorb light at a variety of wavelengths, including near-infrared, visible, and ultraviolet (UV). The nonparamagnetic defect, also recognized as the “*B*_2_ band”, is one of the oldest known defects in amorphous silica, which occurs due to some form of oxygen deficiency in the silica network [51]. The examples of the optically active oxygen-deficiency-related point defects (E’ centers) in the amorphous silica are ≡Si•Si≡ (paramagnetic positively charged oxygen vacancy); ≡Si• (neutral dangling Si bond) centers; neutral (diamagnetic) oxygen vacancy, which comprises a simple oxygen vacancy (≡Si–Si≡); two-fold-coordinated silicon (-O-_•_Si^•^-O-); nonparamagnetic defect (≡Si–Si≡) caused by a singlet–singlet transition; and dicoordinated silicon lone pair (≡Si–O–Si–O–Si≡). In addition, there are oxygen vacancies with a trapped hole or three-fold-coordinated silicons and different variants of diamagnetic ‘ODCs’ (oxygen-deficiency centers) [61,62,63]. Therefore, the large surface area alongside the significant number of surface defects of SiO_2_ NPs may be the reason behind the observed photochemical activity of the synthesized SiO_2_ NPs.

It is worth mentioning that the intensity of the absorption peak is related to the concentration of nanoparticles. However, it appears that the silica content is what causes the peak to shift, since the observed peak may be caused by the interparticle interactions between different sizes of nanoparticles and aggregation of silica nanoparticles. These results agree with the literature [64].

Tauc’s equation was used to calculate the direct optical band gap of SiO_2_ NPs [65]:

(αhν)^1/n^ = A(hν − E_g_) (7)

where A is a constant, E_g_ is the material’s band gap, and exponent n varies depending on the type of transition. For direct allowed transitions, n = 1/2; for indirect allowed transitions, n = 2; for direct forbidden transitions, n = 3/2; and for indirect forbidden transitions, n = 3. Figure 9b depicts the relationship between (αhν)^2^ and (hν) in the case of prepared SiO_2_ NPs.

The E_g_ extracted from Tauc’s plot was found to be 1.95 eV. According to the above-mentioned, this small value of the band gap may also be attributed to the interference of the impurities of Fe and Al oxides on the surface of silica nanoparticles.

### 3.4. UV Photocatalysis Properties

**Figure 10:**
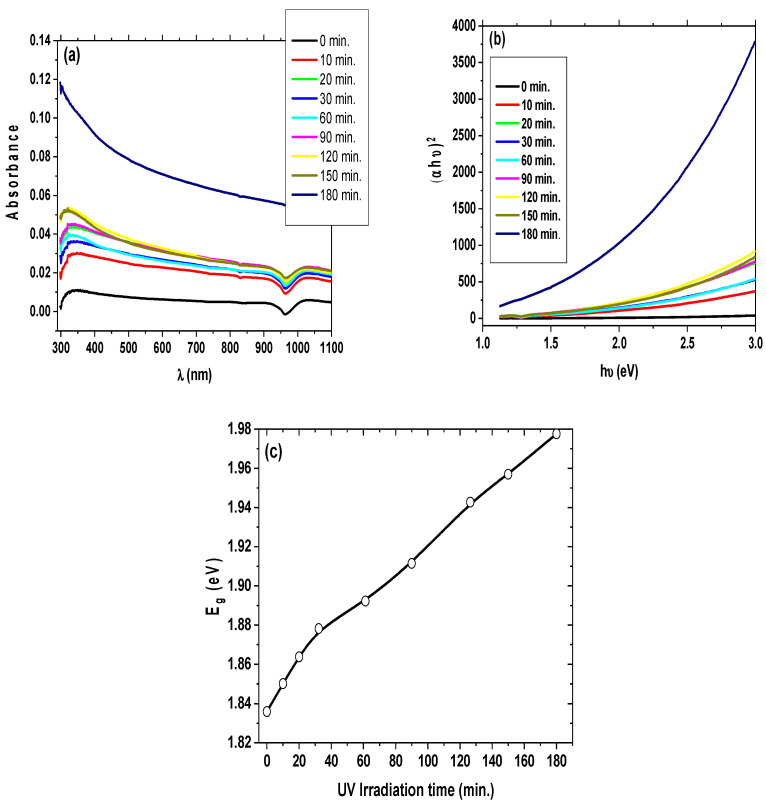
(**a**) Optical absorbance spectrum of the as-prepared silica nanoparticles under the effect of different irradiation times; (**b**) graph of (αhν)^2^ versus (hν) for exposing the prepared silica nanoparticles to UV light under different irradiation times; (**c**) variation of optical band gap energy of the as-prepared silica nanoparticles with different irradiation times.

The effect of UV irradiation time on the structural and optical properties of SiO_2_ NPs was achieved by irradiating a solution of SiO_2_ NPs with λ_max_ of 350 nm; the spectral changes in the UV–Vis region are shown in Figure 10a. Upon 350 nm excitation, the SiO_2_ NPs suspension shows a λ_max_ shift with time. It can be seen from Figure 10a that the absorption onset wavelength of the SiO_2_ NPs is slightly shifted to shorter wavelengths with increasing time irradiation. The maximum shift at ∼350 nm is due to the UV absorption of the SiO_2_ NP suspension. The increasing contribution of different surface structural defects of the SiO_2_ NPs results in a shift in their absorption. Nevertheless, with the increase in the duration of UV irradiation, a slight shift in the absorption peak is observed due to increased particle aggregation. The change in particle aggregation size and the shift due to surface defects with increasing irradiation are a rationale for 350 nm excitation. Due to the initial high concentration of small reactive SiO_2_ NPs that combine to form larger particles, the process begins as a rapid change in size. As the particles coalesce over longer periods, the rate of growth diminishes. Furthermore, this process is feasible due to the absence of a capping agent that would cap the boundaries [66]. Figure 10b displays the band gap of SiO_2_ NPs as determined using the Tauc relation. The energy gap data exhibit a constant increase, indicating that particle aggregates are growing as UV exposure time increases. The fluctuation of the energy band gap with UV exposure time is shown in Figure 10c. Another important factor is the dielectric property of the SiO_2_ NPs, which also plays a major role. The electromagnetic wave interacts with the material being irradiated to cause ionization. The material’s charged particles (SiO_2_ NPs) encounter force, which causes them to further rotate or polarize. The dipolar rotation takes place with the increase in the exposure time of UV irradiation, which provides more time to allow the nanoparticles to aggregate, and absorb more UV photons resulting in more UV/materials (SiO_2_ NPs) interactions. Such interactions with conglomerates of nanoparticles with different sizes lead to distribution in the system and result in different band gap energies [67].

### 3.5. Photodegradation Performance

**Figure 11:**
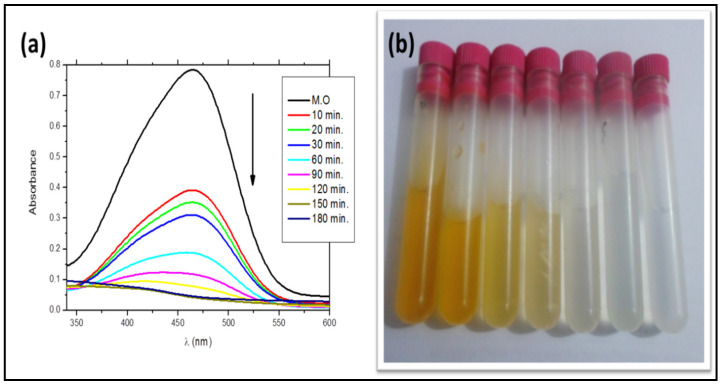
(**a**) UV–Vis absorption spectra for photocatalytic degradation of MO dye by SiO_2_ NPs; (**b**) photographic image for MO dye color removal using SiO_2_ NPs.

**Figure 12:**
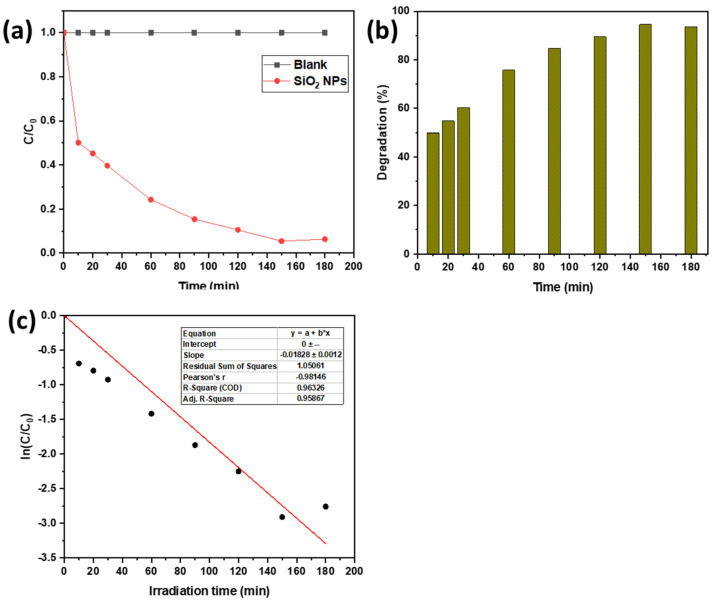
(**a**) Photocatalytic activity for the MO degradation using SiO_2_ NP photocatalyst under UV light irradiation; (**b**) MO photodegradation rate on the surface of SiO_2_ NPs, (**c**) the kinetic relationship of ln(C/C_0_) vs. irradiation time curves.

The UV–Vis spectral change of 50 mg/L MO as a function of irradiation time during photodegradation by the SiO_2_ NPs photocatalyst under UV irradiation is shown in Figure 11a. The obtained data indicated that the reduction in MO dye by SiO_2_ NPs is a function of the exposure time. The intensity was normalized with the initial value at the beginning of irradiation. With time increasing from 0 to 180 min, the intensity of the characteristic peaks of the absorption band at 465 nm progressively decreased with proceeding exposure time, suggesting that the MO dye was gradually photodegraded by the SiO_2_ NP photocatalyst. This was evidenced by the complete decolorization of MO dye within 150 min, which reached approximately ~95% (Figure 11b) and was assured by the obtained absorption spectra. However, when the same experiment was performed without SiO_2_ NPs, an insignificant change in the absorbance peak of MO under these conditions was obtained even after 180 min of UV irradiation, demonstrating that there is no noticeable loss of MO concentration. Moreover, a very slight degradation of about 3.86% in the presence of SiO_2_ NPs and the absence of UV light was obtained when the solution was stirred in the dark for 180 min, which may have been caused by limited dye adsorption to the surface of the SiO_2_ NPs. These findings confirmed that a catalyst and UV radiation are both necessary for effective dye degradation (Figure 12a).

With the addition of SiO_2_ NPs to the MO solution, it was observed that the maximum absorption of the solution decreased over the irradiation time, and the MO color virtually vanished in 150 min. Within 10 min of UV light exposure, photocatalytic performance reached 50%; however, after 150 min of UV light irradiation, MO was degraded photo-catalytically over SiO_2_ NPs at a rate of 95%. The plot of relative MO concentration (C/C_0_) versus irradiation time for SiO_2_ NPs is shown in Figure 12b, where C represents the MO concentration at the irradiation time (t) and C_0_ is the MO concentration prior to UV irradiation. It was found that the SiO_2_ NPs exhibited photocatalytic properties under UV light towards the degradation of MO, as the concentration of the MO solution decreased with increasing time of exposure to UV light.

The SiO_2_ NPs exhibited good performance based on the obtained results of dye degradation. When SiO_2_ NPs are exposed to UV radiation, electrons in the valence band are excited to the conduction band and generate electron–hole pairs. Hydroxyl radicals are produced when the holes in silica nanoparticles interact with water molecules or hydroxide ions. To prolong the recombination of electron–hole pairs during photocatalytic oxidation, oxygen is typically provided as an electron acceptor. The hydroxyl radical is considered a potent oxidizing agent, which destroys organic contaminants present on or near the surface of silica nanoparticles. Thus, the photooxidation of contaminants is proceed according to the following mechanisms: (i) photoabsorption of the SiO_2_ NP catalyst; (ii) the generation of electrons and holes; (iii) the transfer of charge carriers; and (iv) utilization of the charge carriers by the reactants. From the obtained data, it can be concluded that the SiO_2_ NPs prepared from rice husk ash can be used as a powerful photodegradation catalyst for toxic dyes.

The experimental data of kinetic behavior for photocatalytic degradation of MO using SiO_2_ NP photocatalyst were fitted by the first-order relation as follows [68]:

(8) $$\ln\left(\frac{C}{C_{0}}\right)=-\mathrm{Kt}$$

where K is the apparent rate constant (min^−1^), t is the reaction time, C_0_ and C are the concentration of MO dye at 0 and t, respectively. Figure 12c illustrates the linear relation between ln(C/C_0_) and the irradiation time for MO degradation using SiO_2_ NPs. As can be seen in Figure 12c, the photocatalytic degradation curve fits well with the pseudo-first-order kinetic. The apparent reaction rate constant (K) for the photodegradation of MO on the surface of SiO_2_ NPs was estimated to be 0.01828 min^−1^ with an R^2^ value of 0.9632.

### 3.6. Probable Mechanism for Photocatalytic Degradation of MO Dye

Badr et al. reported that when comparing the X-ray photoelectron spectra of SiO_2_ NPs coated with Ag NPs to pure SiO_2_ NPs, a negative shift occurred. They suggested that this shift may be due to an electron transfer between the SiO_2_ NPs and attached metal/metal oxide nanoparticles. Additionally, it was discovered that the SiO_2_ NPs were photoexcited when exposed to UV light and displayed an absorption band at about 309 nm. This is explained by the charge transfer from a bonding orbital of Si-O to a *2p* nonbonded orbital of nonbridging oxygen [24]. According to the literature, oxygen defects or metal/metal oxide nanoparticles on the surface of SiO_2_ would improve the separation of photogenerated electron–hole pairs with a decreasing rate of electron–hole pair recombination, and therefore boost the catalytic activity of SiO_2_-based photocatalyst [69,70]. In this work, the prepared SiO_2_ NPs from the rice husk ash probably contained some impurities such as Fe and Al ions or oxides; thus, the suggested degradation mechanism will involve the effect of SiO_2_ NPs besides the ions/oxides of Fe and Al deposited on the surface of SiO_2_ NPs.

When a UV photon hits the surface of SiO_2_ NPs, photocatalytic active centers will form on the surface of SiO_2_ NPs. This happens when an electron from a material’s valence band jumps to its conduction band, producing a hole with a positive charge in the valence band (h_vb+_) and a charge increase with a negative charge in the conduction band (e_cb−_) (Equation (9)). In turn, this leads to the formation of valence band holes that interact with the chemisorbed H_2_O molecules to produce reactive species such ^•^OH radicals, which attack dye molecules one at a time to degrade them totally (Equation (10)). However, in a few nanoseconds, e_cb−_ and h_vb+_ can combine once more on a particle’s surface, releasing energy as heat. The e_cb−_ and h_vb+_ can interact with species that have been adsorbed to or close to the particle’s surface, which could be trapped in surface states. The superoxide radical anion (O_2_ˉ^•^) is created when the e_cb−_ reacts with an acceptor such as dissolved oxygen to generate ^•^O_2_H (Equations (11) and (12)). In contrast, h_vb+_ and the donor may interact to form a ^•^OH radical from ^-^OH and ^•^O_2_H that attacks the MO (Equation (13)). The efficiency of SiO_2_ NPs is significantly influenced by the amount of ^•^OH radicals, as mentioned before [71]. This means that any factor that promotes the generation of ^•^OH radicals will accelerate the rate of MO photodegradation by the photocatalysis process. Fe^3+^ or Al^3+^ ions are absorbed on the surface of SiO_2_ NPs throughout the process and combine with the electrons in the SiO_2_ NPs’ conduction band to produce the corresponding metal. It lessens the recombination of charges (h_vb+_ and e_cb−_) and encourages the creation of the ^•^OH radical. The oxidative process frequently causes an organic substrate to completely mineralize into CO_2_ and H_2_O. The ability of Fe^3+^ and Al^3+^ to behave as electron scavengers by trapping electrons and creating holes may be the reason behind their enhancing effect (Equations (14)–(16)). The deposited Fe_2_O_3_ and/or Al_2_O_3_ on the surface of SiO_2_ NPs could have an impact on the electron–hole recombination process, which regulates the photocatalytic destruction of MO by SiO_2_ NPs. The deposited Fe_2_O_3_ and/or Al_2_O_3_ on the surface of SiO_2_ NPs are thought to play a large role in the consumption of electrons and their transmission to H^+^ ions or O_2_. As a result, the delay in electron–hole recombination will boost the photocatalytic activity of the SiO_2_ NP photocatalysts, which in turn speeds up the production of hydroxyl radicals and increases the rate at which MO started by ^•^OH is degraded (Equations (17)–(24)) [72,73,74,75,76]:

(9) $${\mathrm{SiO}}_{2}+h\upsilon \to e_{\mathrm{cb}}^{-}+h_{\mathrm{vb}}^{+}$$

(10) $$\left(H_{2}O\to H^{+}+{\mathrm{OH}}^{-}\right)+h_{\mathrm{vb}}^{+}\to H^{+}+{\mathrm{HO}}^{∙}$$

(11) $$O_{2}+e_{\mathrm{cb}}^{-}\to O_{2}^{-∙}$$

(12) $$O_{2}^{-∙}+\left(H^{+}+{\mathrm{HO}}^{-}\right)\to {\mathrm{HO}}_{2}^{∙}+{\mathrm{HO}}^{-}$$

(13) $${\mathrm{HO}}_{2}^{∙}+{\mathrm{HO}}^{-}+h_{\mathrm{vb}}^{-}\to {\mathrm{HO}}^{∙}$$

(14) $$e_{\mathrm{cb}}^{-}+{\mathrm{Fe}}^{3+}\to {\mathrm{Fe}}^{0}$$

(15) $$e_{\mathrm{cb}}^{-}+{\mathrm{Al}}^{3+}\to {\mathrm{Al}}^{0}$$

(16) $$e_{\mathrm{cb}}^{-}+H^{+}\to H_{2}↑$$

(17) $${\mathrm{Fe}}_{2}O_{3}+e_{\mathrm{cb}}^{-}\to {\mathrm{Fe}}_{2}O_{3}\left(e_{\mathrm{cb}}^{-}\right)$$

(18) $${\mathrm{Al}}_{2}O_{3}+e_{\mathrm{cb}}^{-}\to {\mathrm{Al}}_{2}O_{3}\left(e_{\mathrm{cb}}^{-}\right)$$

(19) $${\mathrm{Fe}}_{2}O_{3}\left(e_{\mathrm{cb}}^{-}\right)+H^{+}\to {\mathrm{Fe}}_{2}O_{3}+H_{2}↑$$

(20) $${\mathrm{Al}}_{2}O_{3}\left(e_{\mathrm{cb}}^{-}\right)+H^{+}\to {\mathrm{Al}}_{2}O_{3}+H_{2}↑$$

(21) $${\mathrm{Fe}}_{2}O_{3}\left(e_{\mathrm{cb}}^{-}\right)+O_{2}\to {\mathrm{Fe}}_{2}O_{3}+O_{2}^{-∙}$$

(22) $${\mathrm{Fe}}_{2}O_{3}+O_{2}^{-∙}+\left(H^{+}+{\mathrm{HO}}^{-}\right)\to {\mathrm{HO}}_{2}^{∙}+{\mathrm{HO}}^{-}$$

(23) $${\mathrm{Al}}_{2}O_{3}\left(e_{\mathrm{cb}}^{-}\right)+O_{2}\to {\mathrm{Al}}_{2}O_{3}+O_{2}^{-∙}$$

(24) $${\mathrm{Al}}_{2}O_{3}+O_{2}^{-∙}+\left(H^{+}+{\mathrm{HO}}^{-}\right)\to {\mathrm{HO}}_{2}^{∙}+{\mathrm{HO}}^{-}$$

**Figure 13:**
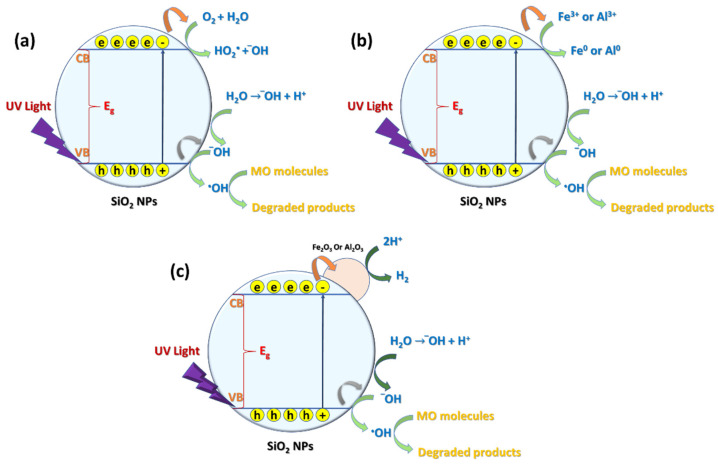
Proposed mechanisms of the photocatalytic effect of (**a**) SiO_2_ NPs, (**b**) SiO_2_ NPs defiled by Fe^3+^ and/or Al^3+^ ions, and (**c**) Fe_2_O_3_ and/or Al_2_O_3_ deposited on the surface of SiO_2_ NPs.

The ability of each Fe^3+^ and/or Al^3+^ ion to consume three (e_cb_^−^) and generate three ^•^OH ions, which are responsible for the degradation of dye, may be a contributing factor in the effective catalysis of the SiO_2_ NPs coated with Fe^3+^ and/or Al^3+^ ions, while Fe_2_O_3_ and/or Al_2_O_3_ deposited on the surface of SiO_2_ NPs act as electron–hole separation centers. Since SiO_2_ has a higher Fermi level than Fe_2_O_3_ and/or Al_2_O_3_, it is possible thermodynamically for electrons to pass from the SiO_2_ NP conduction band to Fe_2_O_3_ and/or Al_2_O_3_ at the interface. As a result, the Schottky barrier was generated at the contact zone between the Fe_2_O_3_ or Al_2_O_3_ and the SiO_2_ NPs, which enhances charge separation and improves the photocatalytic activity of SiO_2_ NPs. The Fe_2_O_3_ and/or Al_2_O_3_ boost the activity of SiO_2_ NPs because there is an energy difference between the valance and conduction bands of Fe_2_O_3_ and/or Al_2_O_3_ and those of SiO_2_ NPs [77,78,79,80,81]. Figure 13 shows the predicted mechanisms of the photodegradation process, which are implemented by the hydroxide attack.

**Figure 14:**
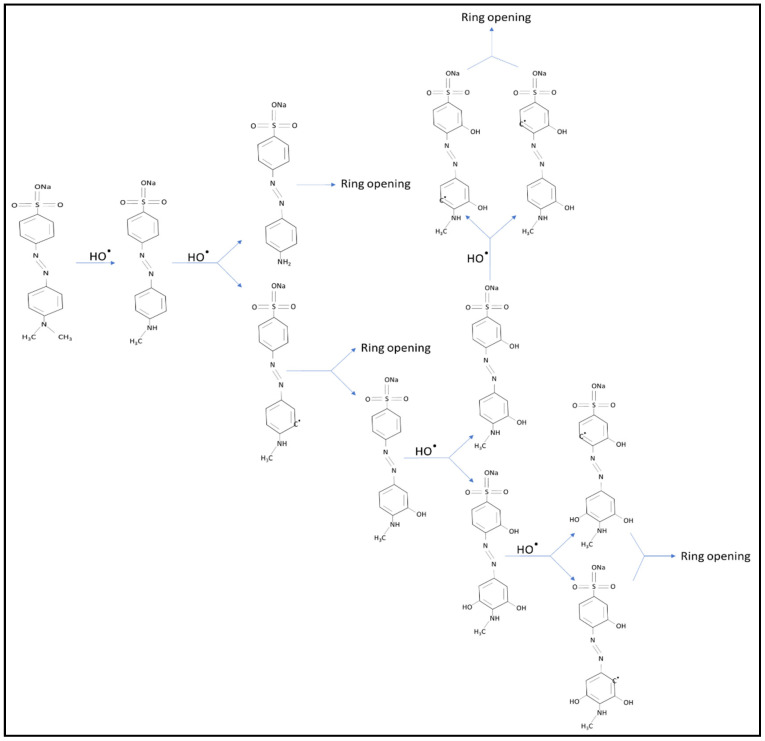
Illustrating the mechanism of the potential pathways for the photocatalytic degradation of MO by SiO_2_ NPs, SiO_2_ NPs defiled by Fe^3+^ and/or Al^3+^ ions, or Fe_2_O_3_ and/or Al_2_O_3_ deposited on the surface of SiO_2_ NPs.

Figure 14 shows a schematic diagram that summarizes the proposed steps assumed for the interaction of hydroxide radicals with MO or intermediate photoproducts. Thus, the decolorization mechanism of the MO dye is mainly caused by the hydroxide attack.

### 3.7. Comparative Study of Photocatalytic Degradation of Synthesized SiO_2_ NPs with Those from the Literature

Table 4 provides an overview of several literature results on the photocatalytic degradation of dyes utilizing different systems-based SiO_2_ NPs and current synthesized SiO_2_ NPs. According to the comparative investigation, the synthesized SiO_2_ NPs exhibited an effective photocatalytic activity.

### 3.8. Antibacterial Behavior

**Figure 15:**
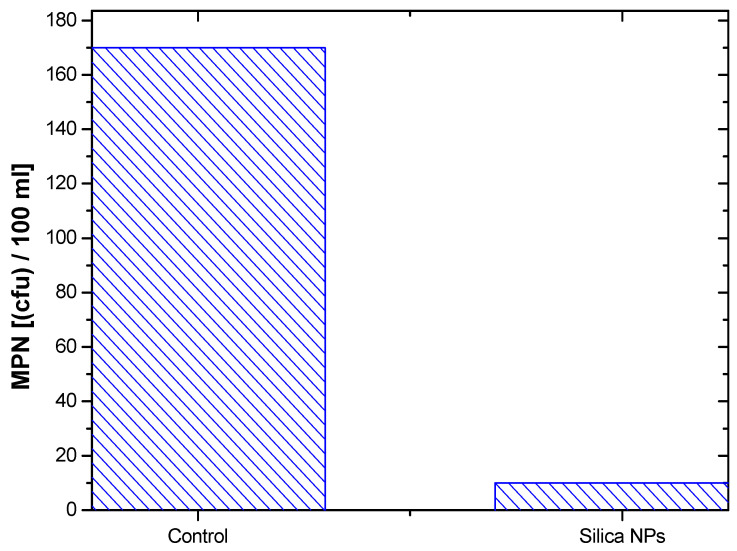
Antibacterial activity of the prepared silica nanoparticles against coliform bacteria as estimated by the MPN method.

As shown in Figure 15, the MPN of coliform bacteria before and after treatment by SiO_2_ NPs was estimated to be 170 CFU/100 mL and 10 CFU/100 mL for untreated and treated samples, respectively, giving a bacterial growth inhibition of 94.12%. The generation of reactive oxygen species (ROS), electrostatic interaction, and attached Fe^3+^/Al^3+^ species all play major roles in the antibacterial activity of SiO_2_ NPs. As a result, it is anticipated that the area around the SiO_2_ NPs and the bacterial aggregation will be enriched by the active antimicrobial species, such as Fe^3+^/Al^3+^ ions and ROS, where the efficient discharge of Fe^3+^/Al^3+^ ions might be supported by the porous structure of SiO_2_ NPs. These mechanisms can cause damage at many levels. Additionally, SiO_2_ NPs or Fe^3+^/Al^3+^ ions can attach to the constitutive proteins of the cell membrane, which are involved in transmembrane adenosine triphosphate (ATP) production, causing membrane damage and cellular content leakage.

## 4. Conclusions

In this study, a sol–gel method was employed to synthesize silica nanoparticles from rice husk ash as a low-cost agricultural waste. Different characterization techniques were used to demonstrate the successful preparation of SiO_2_ NPs, such as XRD, SEM, EDX, TEM, AFM, BET, and CA analyzers. The obtained findings indicated that the prepared SiO_2_ NPs were fabricated in an amorphous phase and spherical shapes with sizes of 60–80 nm. The elemental analysis confirmed that the composition of the prepared sample consists predominately of SiO_2_. In addition, the SiO_2_ NPs showed a surface area of 78.52 m^2^/g. Then, the synthesized SiO_2_ NPs were examined as a photocatalyst to degrade the MO dye in the aqueous solutions under UV irradiation. The photocatalytic results revealed that about 50% of MO dye was degraded after 10 min, while 95% of dye degradation was achieved within 150 min. Moreover, the SiO_2_ NPs exhibited an antibacterial effect against coliform bacteria present in the surface waters, which achieved a bacterial growth inhibition of 94.12% within 90 min.

## Figures and Tables

**Table 2:** BET surface area, BJH cumulative surface area, and total pore volume of SiO_2_ NPs sample.

Parameter	SiO_2_ NPs
BET surface area (m^2^/g)	78.52
BJH adsorption cumulative surface area (m^2^/g)	40.63
BJH desorption cumulative surface area (m^2^/g)	38.48
Total pore volume (cm^3^/g)	0.062
Average pore diameter (nm)	3.158

**Table 3:** The results of wettability properties of SiO_2_ NPs.

Statistics	Time [s]	CA Left [°]	CA Right [°]	CA Mean [°]	ST [mN/m]	Y-L β	Y-L r [mm]
Mean	5.00318	159.15092	157.67678	158.41385	76.633084	−0.3450381	1.6443942
Std deviation	2.91972	2.5912125	0.2020522	1.3742527	1.3486686	0.0065737	0.0014165
Min	0	156.24350	157.23423	156.80051	71.624433	−0.3690024	1.6402470
Max	10.0059	175.76149	158.23625	166.99887	79.423913	−0.3313800	1.6515908

**Table 4:** Comparison of photocatalytic performances of the synthesized SiO_2_ NPs from RHA and systems-based SiO_2_ NPs and different photocatalysts for dye degradation at different photodegradation times.

Sr. No.	Photocatalyst	Pollutant	Light Source	Degradation Time	Photocatalytic Activity	Ref.
1	SiO_2_ Ag NPs@SiO_2_ NPs Au NPs@SiO_2_ NPs Au NPs&Ag NPs@SiO_2_ NPs Ag^+^@SiO_2_ NPs Au^3+^@SiO_2_ NPs	Methyl red	Xe Lamp	120 min 40 min 40 min 35 min 35 min 10 min	100%	[24]
2	SiO_2_	Methylene blue, Methyl orange	Hg Lamp	90 min	99.51%	[69]
3	SiO_2_ NPs (RHA)	Methyl red	Sunlight	120 min	95%	[82]
4	SiO_2_ NPs SiO_2_@Ag NPs SiO_2_@Au NPs	Methyl orange	Xe Lamp	120 min 65 min 85 min	100%	[83]
5	Chitosan Silica Composite	Methyl orange	Sunlight	70 min	94.01%	[84]
6	[FemIL@SiO_2_@ Mag]_2_MoO_4_	Methyl orange	Hg Lamp	30 min	99%	[85]
7	SiO_2_ (RHA)	Methyl orange	Hg Lamp	150 min	~95%	Present work
8	ZnO nanorod ZnO nanospindle ZnO nanoflower	Methyl orange	Hg Lamp	180 min	89% 80% 69%	[86]
9	ZnO nanoparticle	Methyl orange	Hg Lamp	80 min	100%	[87]
10	ZnO-Sn/GO nanocomposites	Methyl orange	Xe Lamp	120 min	96.2%	[88]
11	TiO_2_ nanoparticles	Methyl orange	Xe Lamp	240 min	67.12%	[89]
12	TiO_2_ nanoparticles	Methyl orange	Sunlight	30 min	60%	[90]
13	Zr and Ag codoped TiO_2_ nanoparticles	Methyl orange	Tungsten lamp	7 min	100%	[91]
14	TiO_2_ Sachtopore (NiSO_4_/TiO_2_ = 0.2%) nanoparticles Pure TiO_2_ Sachtopore TiO_2_ Sachtopore (NiSO_4_/TiO_2_ = 0.1%) nanoparticles	Methyl orange	UV Lamp	120 min	45% 38% 20%	[7]

## Data Availability

The data supporting the findings of this study are available within the article.
